# ASCL1 regulates neurodevelopmental transcription factors and cell cycle genes in brain tumors of glioma mouse models

**DOI:** 10.1002/glia.23873

**Published:** 2020-06-23

**Authors:** Tou Yia Vue, Rahul K. Kollipara, Mark D. Borromeo, Tyler Smith, Tomoyuki Mashimo, Dennis K. Burns, Robert M. Bachoo, Jane E. Johnson

**Affiliations:** ^1^ Department of Neuroscience University of Texas Southwestern Medical Center Dallas Texas USA; ^2^ McDermott Center for Human Growth and Development University of Texas Southwestern Medical Center Dallas Texas USA; ^3^ Department of Neurology and Neurotherapeutics University of Texas Southwestern Medical Center Dallas Texas USA; ^4^ Department of Internal Medicine University of Texas Southwestern Medical Center Dallas Texas USA; ^5^ Department of Pathology University of Texas Southwestern Medical Center Dallas Texas USA; ^6^ Department of Pharmacology University of Texas Southwestern Medical Center Dallas Texas USA

**Keywords:** ASCL1, brain tumor, glioma development, mouse model, transcription factor function

## Abstract

Glioblastomas (GBMs) are incurable brain tumors with a high degree of cellular heterogeneity and genetic mutations. Transcription factors that normally regulate neural progenitors and glial development are aberrantly coexpressed in GBM, conferring cancer stem‐like properties to drive tumor progression and therapeutic resistance. However, the functional role of individual transcription factors in GBMs in vivo remains elusive. Here, we demonstrate that the basic‐helix–loop–helix transcription factor ASCL1 regulates transcriptional targets that are central to GBM development, including neural stem cell and glial transcription factors, oncogenic signaling molecules, chromatin modifying genes, and cell cycle and mitotic genes. We also show that the loss of ASCL1 significantly reduces the proliferation of GBMs induced in the brain of a genetically relevant glioma mouse model, resulting in extended survival times. RNA‐seq analysis of mouse GBM tumors reveal that the loss of ASCL1 is associated with downregulation of cell cycle genes, illustrating an important role for ASCL1 in controlling the proliferation of GBM.

## Main Points

- ASCL1 is coexpressed with neural stem cell/glial transcription factors in GBM.
- ASCL1 binds to genes that are important for cell proliferation and cancer in brain tumors.
- Loss of ASCL1 downregulates cell cycle genes and increase survival of glioma mouse model.

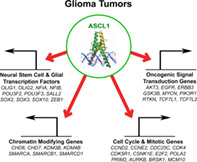

## 1 INTRODUCTION

Glioblastomas (GBMs) are incurable brain tumors most commonly found in adults. Despite significant advances in imaging and surgical resection techniques combined with aggressive radiotherapy and chemotherapy, the median survival for GBM patients remains stagnated between 14 and 16 months, with greater than 90% of patients succumbing to their disease within 5 years of diagnosis (Ostrom et al., 2016). A major reason for this poor prognosis is due to the high degree of heterogeneity and plasticity of these neoplasms, and the lack of mechanistic insights into the pan‐therapeutic resistance of GBM tumor cells (Babu et al., 2016; Brennan et al., 2013; Lathia, Heddleston, Venere, & Rich, 2011).

Concerted sequencing efforts from the Cancer Genome Atlas (TCGA) Research Network revealed a complex somatic landscape for GBMs involving oncogenes (*BRAF*, *EGFR*, *PDGFRα*, *MET*, *PIK3C*, *MYCN*), tumor suppressor genes (*CDNK2A/B*, *PTEN*, *NF1*, *RB1*) and chromatin modifying genes, which converge to activate signaling pathways (pAKT, Ras/MAPK, STAT) to promote tumor proliferation and growth (Brennan et al., 2009, 2013; TCGA, 2008; Verhaak et al., 2010). Emerging evidence also suggests that a cellular hierarchy may exist within the heterogeneous GBM tumor composition, where a subpopulation of quiescent cancer stem‐like cells, or glioma stem cells (GSCs), are postulated to be responsible for driving tumor growth, progression, and the development of resistance to therapeutic treatments (Bao et al., 2006; Chen et al., 2012; Lan et al., 2017; Lathia, Gallagher et al., 2011; Lathia, Heddleston, et al., 2011; Parada, Dirks, & Wechsler‐Reya, 2017). Despite displaying an aberrant array of mutations, GSCs are universally marked by coexpression of a combination of transcription factors, some of which include ASCL1, NFIA, NKX2.2, OLIG2, POU3F2, SALL2, SOX2, and ZEB1 (Glasgow et al., 2017; Lu et al., 2016; Rheinbay et al., 2013; Singh et al., 2017; Suva et al., 2014). These transcription factors have been extensively studied in the developing central nervous system (CNS), where each has been shown to regulate the fate, proliferation and/or migration of neural progenitor and glial precursor cells in stage specific processes. In the context of gliomas, these transcription factors are often constitutively coexpressed and have been shown to function in a combinatorial manner in determining the tumorigenicity and differentiation status of tumor cells (Gangemi et al., 2009; Ligon et al., 2007; Rheinbay et al., 2013; Singh et al., 2017; Suva et al., 2014).

In this study, we focus on ASCL1, a class II basic‐helix–loop–helix (bHLH) transcription factor that forms a heterodimer with class I bHLH E‐proteins (such as E47/TCF3) to activate specific target genes (Kageyama, Ohtsuka, Hatakeyama, & Ohsawa, 2005). During embryogenesis, ASCL1 is expressed in specific populations of neural progenitor domains and glial precursor cells throughout the neural tube from the spinal cord to the brain (Helms et al., 2005; Parras et al., 2004, 2007; Sugimori et al., 2007, 2008; Vue, Kim, Parras, Guillemot, & Johnson, 2014), including in neurogenic regions of the adult brain (Kim, Ables, Dickel, Eisch, & Johnson, 2011; Kim, Leung, Reed, & Johnson, 2007). Recently, ASCL1 was shown to be capable of reorganizing and promoting the accessibility of closed chromatin in embryonic stem cells, neural progenitors, as well as glioma cell lines (Casey, Kollipara, Pozo, & Johnson, 2018; Park et al., 2017; Raposo et al., 2015). Not surprisingly, genome wide profiling revealed a critical role for ASCL1 in interacting with both Wnt and Notch signaling pathways to control the tumorigenicity of glioma cells in culture (Park et al., 2017; Rajakulendran et al., 2019; Rheinbay et al., 2013). To date however, whether ASCL1 is absolutely required for glioma tumor development in the brain as it has been shown for a mouse model of small cell lung carcinoma (SCLC) (Borromeo et al., 2016) remains to be determined. Here, we sought to identify the direct in vivo role and transcriptional targets of ASCL1 in brain tumors of previously characterized patient‐derived xenograft (PDX)‐GBM (Marian et al., 2010; Marin‐Valencia et al., 2012) and a genetically engineered glioma mouse model (Lin et al., 2004; Zhu et al., 2001).

## 2 MATERIALS AND METHODS

### 2.1 Glioma mouse models

PDX‐GBM (R738 and R548) were passaged orthotopically in the brains of NOD‐SCID mice as previously described (Marian et al., 2010; Marin‐Valencia et al., 2012). Generation and genotyping of mouse strains used to generate the glioma models were as previously reported: *Glast* ^*CreERT2*^ knock‐in (Mori et al., 2006); *Ascl1* ^*GFP*^ knock‐in [Ascl1^tm1Reed^/J 012881] (Kim et al., 2007); *Ascl1* ^*F*^ [Ascl1‐floxed] (Andersen et al., 2014; Pacary et al., 2011); *Nf1* ^*F*^ [Nf1^tm1Par^/J 017639] (Zhu et al., 2001); *Tp53* ^*F*^ [Tp53‐floxed] (Lin et al., 2004); and the Cre reporter lines *R26R* ^*LSL‐YFP*^ [Gt(ROSA)26Sor^tm1(EYFP)Cos^/J 006148] (Srinivas et al., 2001) and *R26R* ^*LSL‐tdTOM*^ [Gt(ROSA)26Sortm^14(CAG‐tdTomato)Hze^/J 013731] (Madisen et al., 2010). All animal procedures followed NIH guidelines and were approved by the UT Southwestern Institutional Animal Care and Use Committee.

### 2.2 Mouse breeding and tamoxifen administration

The appearance of a vaginal plug was considered embryonic day (E) 0.5, and the day of birth was noted as postnatal day (P) 0. To induce tumor formation in the brains of *Glast* ^*CreERT2/+*^ *;Nf1* ^*F/F*^ *;Trp53* ^*F/F*^ mice, tamoxifen (Sigma T5648, dissolved in 10% ethanol/90% sunflower oil) was administered intraperitoneally (62.5 mg/kg body weight) to pregnant females at E14.5. Due to the effects of tamoxifen on birth complications, cesarean section was performed and pups were carefully introduced and raised by a foster female.

### 2.3 Tissue preparation, H&E staining, and immunofluorescence

Tumor bearing mice were trans‐cardiac‐perfused with 4% PFA in PBS. Brains were submerged in 30% sucrose/PBS at 4°C, and embedded in O.C.T. for cryosectioning. H&E staining of tumors was done by the UT Southwestern Histopathology Core. Grading of brain tumors was determined by a board certified neuropathologist.

For immunohistochemistry, tissue sections were incubated with primary antibody in 1% goat or donkey serum/0.3% Triton X‐100/PBS overnight, followed by incubation with secondary antibody conjugated with Alexa Fluor 488, 568 or 647 (Molecular Probes), and coverslipped with Vectashield (#101098‐042) for confocal microscopy (LSM 510 and 720). The following antibodies were used:

**Table:** 

Primary antibodies	Source and catalogue number	Dilution
Chicken anti‐GFP	Chemicon, AB16901	1:500
Goat anti‐SOX10	R&D Systems, AF2864	1:20
Guinea pig anti‐ASCL1	Kim et al. (2008) TX518	1:1,000–1:10,000
Mouse anti‐GFAP	Sigma, G3893	1:500
Mouse anti‐MBP	Calbiochem, NE1019	1:300
Mouse anti‐NEUN	Chemicon, MAB377	1:1,000
Rabbit anti‐Ki67	Abcam, ab15580	1:500
Rabbit anti‐OLIG2	Millipore, AB9610	1:1,000
Rabbit anti‐SOX2	Millipore, AB5603	1:1,000
Rat anti‐PDGFRα (APA5)	BD Pharmingen, 558774	1:100

### 2.4 ChIP‐seq, RNA‐seq, and data analysis

Two independent ASCL1 ChIP‐seq experiments were performed using PDX‐GBMs (R548 and R738) dissected from brains of NOD‐SCID mice exhibiting symptoms of the presence of tumor. Briefly, as previously described (Borromeo et al., 2016), tumor tissues were homogenized and fixed in 1% formaldehyde to crosslink proteins and DNA, followed by quenching with 0.125 M of glycine. Nuclear chromatin was pelleted, washed with cold PBS, and sonicated into 200–300 bp fragments using a Biorupter (Diagenode). A 10% portion of the sheared chromatin was set aside as input DNA. Approximately 100 μg was subjected to immunoprecipitation using ~5 μg of mouse anti‐ASCL1 (Mash1) antibody (BD Biosciences, 556604). Washes and reverse‐crosslinking were performed using Dynabeads Protein G to elute ChIP DNA.

For RNA‐seq experiments, the brain tumors were carefully dissected to enrich for tumor tissues and total RNA was extracted using a Direct‐zol RNA MiniPrep Kit (Zymo Research). RNA integrity number (RIN) for all tumors was determined to be between 8 and 10 using a Bioagilent Analyzer. ChIP DNA and input DNA from PDX‐GBMs and total RNAs from mouse brain tumors were sent for library preparation and sequencing on an Illumina High‐Seq 2000 at the UT Southwestern Next Generation Sequencing Core.

To analyze ASCL1 ChIP‐seq data (GSE152401), sequence reads were aligned to the human reference genome (hg19) using bowtie2 (v.2.2.6) (Langmead & Salzberg, 2012). Low‐quality reads and duplicate reads were removed from aligned files using “samtools view ‐bh‐F 0 × 04 ‐q 10” (v1.2) (Li, 2011) and “Picard MarkDuplicates.jar” (v. 1.131) commands (Picard 2018, Broad Institute, GitHub repository). The ChIP‐seq signal enriched regions were identified using the “findPeaks” module available in HOMER software (v.4.7) (Heinz et al., 2010). The ChIP‐seq signal shown in UCSC browser tracks are normalized read counts. De novo motif discovery and analysis were performed using “findMotifsGenome” module available in HOMER software (v.4.7). A 150 bp region around the peak summit was used to identify the primary binding motif and other potential DNA‐binding co‐Lanfactor motifs.

To analyze mouse tumor RNA‐seq data (GSE152401), sequenced reads were aligned to the mouse mm10 genome using TopHat 2.1.0 (Kim et al., 2013). Default settings were used, with the exception of –G, specifying assembly to the mm10 genome, −‐library‐type fr ‐first strand, and –no‐novel‐juncs, which disregards noncanonical splice junctions when defining alignments. DESeq2 (Love, Huber, & Anders, 2014) was used to incorporate RNA‐seq data from the five biological replicates for *Ascl1* ^*WT*^ and *Ascl1* ^*CKO*^ tumor samples, and differentially expressed genes were identified using default parameters.

To investigate the similarity/difference between *Ascl1* ^*WT*^ and *Ascl1* ^*CKO*^ tumors in comparison to each other and to CNS cell types, multidimensional scaling (MDS) was performed using the plotMDS function available in edgeR package (Robinson, McCarthy, & Smyth, 2010). Finally, to identify enrichment of gene signature sets in rank ordered gene lists obtained from *Ascl1* ^*WT*^ and *Ascl1* ^*CKO*^ tumor samples, gene set enrichment analysis (GSEA) (Subramanian et al., 2005) was performed and the signal‐to‐noise ratio metric was used to rank the genes.

### 2.5 GBM subtype classification and heatmap clustering analyses

The GBM subtype signatures defined by Verhaak et al. (2010) were used for hierarchical clustering for 164 GBM patient samples and five normal brains from TCGA for which RNA‐seq data was available (Brennan et al., 2009, 2013; TCGA, 2008; Verhaak et al., 2010). Spearman rank order correlation and ward.D2 clustering method were applied to identify the various GBM subtypes. Heatmaps were generated using absolute expression values (RPKM) for the selected list of genes or significantly changed genes, and hierarchical clustering was performed using the correlation distance metric and the ward.D2 method using the heatmap.2 function available in the *gplots* R package.

### 2.6 Gene targets and pathway enrichment analysis

To identify ASCL1 putative targets, genes associated with the ASCL1 ChIP‐seq peaks were annotated using GREAT v3.0.0 (http://great.stanford.edu/public/html/) (McLean et al., 2010), which was then cross‐referenced with the top 10% of genes (2,136) whose expression positively correlates with ASCL1 expression by computing the Spearman rank order correlation (>0.4) using RNA‐seq of TCGA GBM expression data. An overlap of 1,106 genes was identified as ASCL1 putative target genes. These genes were then subjected to pathway enrichment analysis performed using ConsensusPathDB (http://cpdb.molgen.mpg.de/) (Herwig, Hardt, Lienhard, & Kamburov, 2016). Relevant significantly enriched over‐represented gene sets were selected for illustration.

### 2.7 Quantification of ASCL1+, OLIG2+, SOX2+, and Ki67+ tumor cells

The number of DAPI+ tumor cells that were ASCL1+ along with each of the various markers were quantified using Image J on 20× immunofluorescence confocal images of both R548 and R738 PDX‐GBMs. Quantifications were performed on at least three images taken from different areas per tumor for each marker (*N* = 4).

To determine the expression of ASCL1, OLIG2, and SOX2 in human GBMs, RNA‐seq of 164 TCGA primary GBM and five normal brain samples (Brennan et al., 2013) were analyzed and categorized into the various subtypes using the 840 GBM Subtype Signature Genes (Verhaak et al., 2010). Average RPKM for *ASCL1*, *OLIG2*, and *SOX2* was determined for each GBM subtype. Outlier samples exhibiting an RPKM value >2 *SD*s away from the mean for each subtype were excluded.

To compare the Ki67 index between *Ascl1* ^*WT*^ (*N* = 6) or *Ascl1* ^*CKO*^ (*N* = 5) tumors, 20× immunofluorescence confocal images were taken from three different areas per tumor. Because the distribution of Ki67+ cells is not uniform within a large growing tumor, we limited our imaging to only regions with the highest density of Ki67+ cells. Quantification of the number of Ki67+;DAPI+/total DAPI+ cells was then performed blind of genotype for each image and compiled for comparison between *Ascl1* ^*WT*^ or *Ascl1* ^*CKO*^ tumors using a Wilcox test.

## 3 RESULTS

### 3.1 Neurodevelopmental transcription factors ASCL1, OLIG2, and SOX2 are highly coexpressed in human GBMs

**FIGURE 1:**
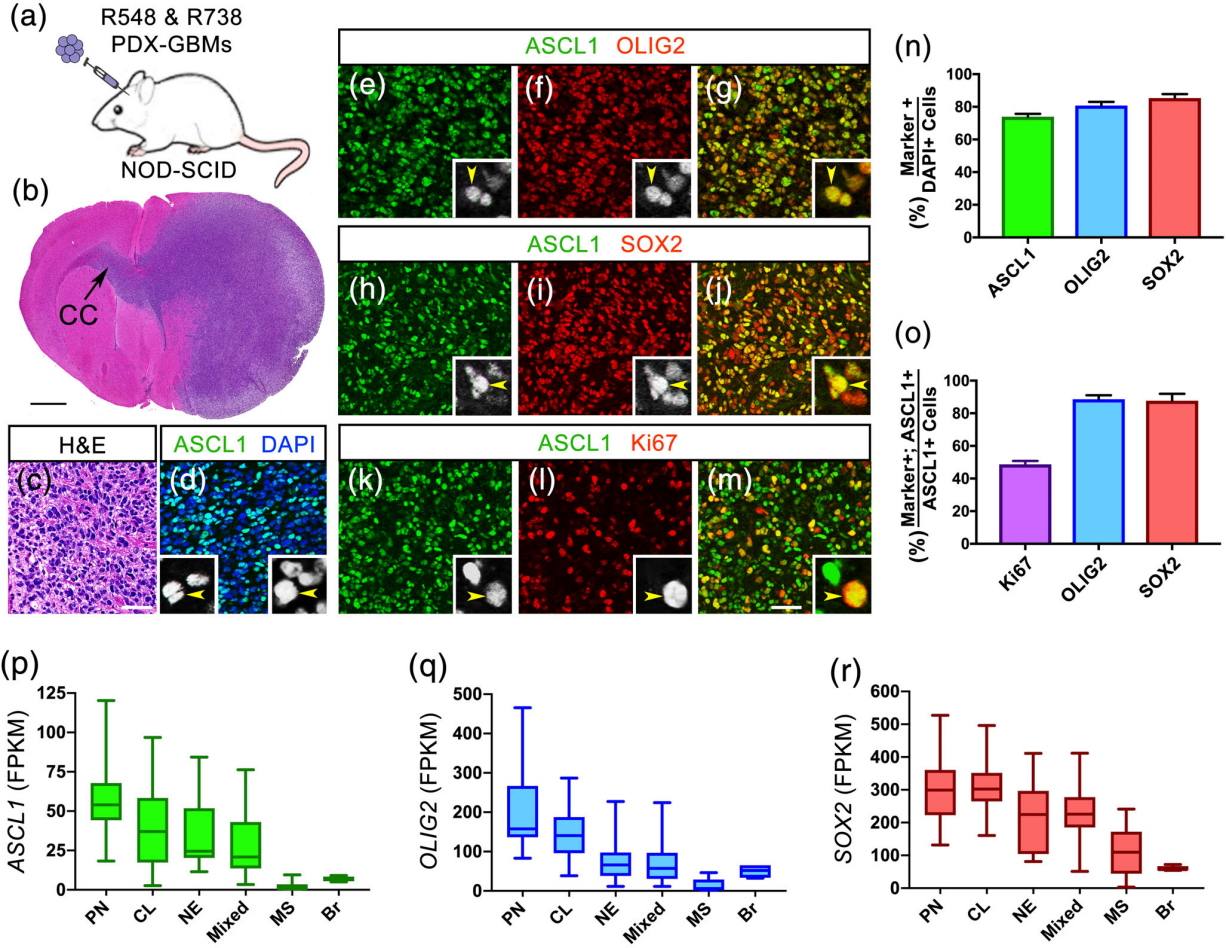
Neurodevelopmental transcription factors ASCL1, OLIG2, and SOX2 are highly expressed in the majority of GBMs. (a) Schematic of PDX‐GBMs (R548 and R738) grown orthotopically in the brains of NOD‐SCID mice. (b and c) H&E staining showing tumor is a high‐grade glioma and is migrating across the corpus callosum (CC). (d–m) Immunofluorescence showing coexpression of ASCL1 with OLIG2 (e‐g), SOX2 (h‐j), and Ki67 (k‐m) in the PDX‐GBMs. (n and o) Quantification of the percentage of DAPI+ tumor cells that are ASCL1+, OLIG2+, or SOX2+ (n), and the percentage of ASCL1+ tumor cells that are also Ki67+, OLIG2+, or SOX2+ (n). *N* = 4 PDX‐GBM. (p–r) Box whisker plot of RNA‐seq data from 164 TCGA Primary GBMs and 5 normal brain samples (Brennan et al., 2013) demonstrating that *ASCL1* (p), *OLIG2* (q), and *SOX2* (r) are highly expressed in the majority of GBM subtypes but are low in MS subtype and normal brain (Br). GBM subtype was determined using the 840 GBM Subtype Signature Genes (Verhaak et al., 2010). CL, classical; MS, mesenchymal; NE, neural; PN, proneural. Mixed GBM subtype express multiple subtype signatures. Scale bar is 1 mm for (b) and 50 μm for (c–m), and 12.5 μm for all insets in (d–m) [Color figure can be viewed at wileyonlinelibrary.com]

ASCL1, OLIG2, and SOX2 have previously been reported to be expressed in GBMs (Gangemi et al., 2009; Ligon et al., 2007; Lu et al., 2016; Park et al., 2017; Rheinbay et al., 2013; Singh et al., 2017; Somasundaram et al., 2005). However, the extent to which these factors are coexpressed in GBM tumors in vivo remains unclear. Using PDX‐GBM lines (R548, R738), in which tumors from patients were passaged orthotopically in the brains of NOD‐SCID mice (Figure 1a) (Marian et al., 2010; Marin‐Valencia et al., 2012), we demonstrated that the transplanted tumors exhibit pathological characteristics of high‐grade gliomas (Figure 1b,c) and express ASCL1, OLIG2, and SOX2 in the majority of tumor cells (Figure 1d–m). Quantification shows that each transcription factor occupied 74%, 81%, and 85% of tumor cells counterstained with DAPI, respectively (Figure 1n). Colocalization analysis revealed that 48% of ASCL1+ tumor cells were positive for the proliferation marker Ki67 (Figure 1k–m,o), whereas over 90% of ASCL1+ cells were OLIG2+ and SOX2+ (Figure 1o), indicating that these three transcription factors are coexpressed in the majority of the PDX‐GBM cells in vivo.

We next sought to determine the expression level of ASCL1, OLIG2, and SOX2 across primary GBMs exhibiting a variety of genomic alterations. Leveraging RNA‐seq data of 164 TCGA primary GBMs, along with five normal control brain samples (Brennan et al., 2013), we first classified these primary GBMs into the four GBM subtypes (proneural, neural, classical, mesenchymal) as previously defined using an 840 gene list (Verhaak et al., 2010). Notably, while 107 samples can be classified into one of the four GBM subtypes, the remaining 57 samples expressed signatures of more than one subtype, which we referred collectively to as mixed GBMs (Figure S1). This finding echoes previous reports demonstrating the presence of multiple GBM subtype identities in different regions or cells of the same GBM tumors (Patel et al., 2014; Sottoriva et al., 2013). Expression of *ASCL1*, *OLIG2*, and *SOX2* across these GBM subtypes showed that they were highest in the proneural and classical subtypes, intermediate in the neural and mixed subtypes, but were extremely low in the mesenchymal subtype, even in comparison to normal brain (Figure 1p–r). Collectively, these findings illustrate that *ASCL1*, *OLIG2*, and *SOX2* are coexpressed at relatively high levels in the majority of primary GBMs with the exception of the mesenchymal subtype.

### 3.2 ASCL1 binds to genes encoding neurodevelopmental and glial transcription factors, oncogene signaling molecules, and factors involved in cell cycle control and chromatin organization

**FIGURE 2:**
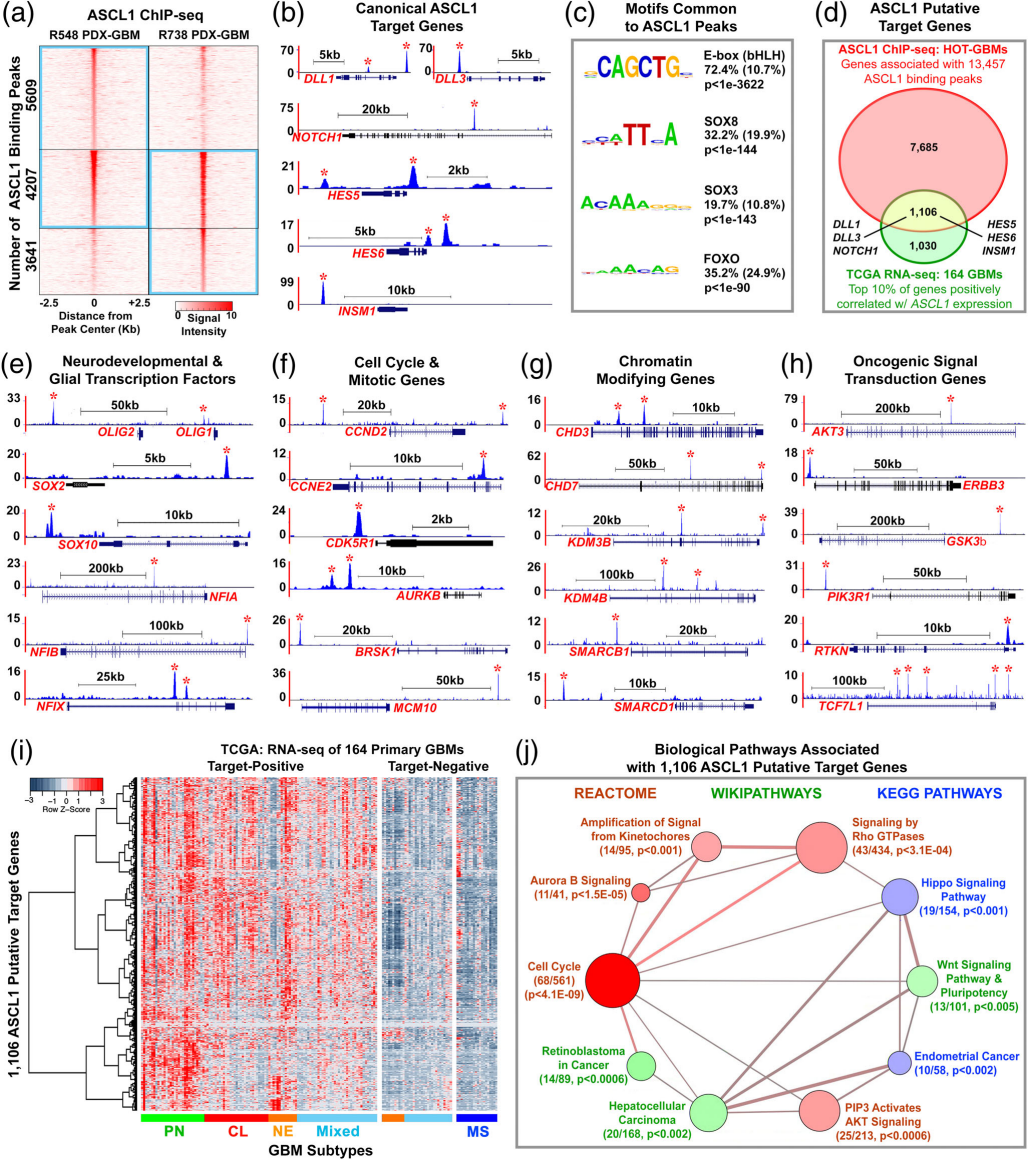
ASCL1 binds to target genes in GBMs involved in glial development, cell cycle progression, and cancer. (a) Heatmap of ASCL1 ChIP‐seq signal intensity ±2.5 kb around 13,457 combined peaks identified in the genome of the PDX‐GBMs. Blue rectangles indicate statistically significant peaks called by Homer. See Supporting Information Table S1 for genomic coordinates of the ASCL1 binding sites. (b) ChIP‐seq tracks of genomic regions surrounding canonical ASCL1 target genes *DLL1*, *DLL3*, *NOTCH1*, *HES5*, *HES6*, and *INSM1*. Asterisks indicate ASCL1 binding peaks meeting statistical criteria. (c) De novo motif analysis shows enrichment of bHLH E‐box, SOX, and FOXO motifs directly beneath ASCL1 binding peaks. (d) Venn diagram of intersecting genes (8,791, red oval) associated with ASCL1 binding peaks in the PDX‐GBMs with the top 10% of genes (2,136, green oval) positively correlated (Spearmann corr < 0.4) with *ASCL1* expression using RNA‐seq data of 164 TCGA GBM samples. The overlap of 1,106 genes (yellow area) defines ASCL1 target genes, which included all the canonical ASCL1 target genes. See Supporting Information Tables [Link], [Link]. (e–h) ChIP‐seq tracks of ASCL1 binding peaks at loci of neurodevelopmental and glial transcription factors (e), cell cycle and mitotic genes (f), chromatin modifying genes (g), and oncogenic signal transduction genes (h). (i) Heatmap and dendrogram illustrating relative expression of 1,106 ASCL1 putative target genes in GBM subtypes using RNA‐seq of 164 TCGA primary GBM samples (Brennan et al., 2013). Note that ASCL1 target‐positive GBMs include all subtypes except mesenchymal, while ASCL1 target‐negative GBMs include all mesenchymal and some neural and mixed GBM subtypes. (j) Gene set over‐representation analysis of 1,106 ASCL1 putative‐target genes using ConsensusPathDB (cpdb.molgen.mpg.de). Biologically relevant enriched pathways are illustrated. Size of circle indicates the number of genes per pathway, size of edge indicates degree of gene overlaps between the pathways, and color indicates database sources. The number of ASCL1 putative‐target genes over‐represented in each pathway, and respective *p*‐value are indicated. See Supporting Information Table S5 for complete gene set over‐representation analysis [Color figure can be viewed at wileyonlinelibrary.com]

Chromatin immunoprecipitation followed by deep sequencing (ChIP‐seq) has previously been performed for ASCL1 in glioma cell lines in culture, and a dual role for ASCL1 was proposed to either promote or attenuate tumorigenicity depending on context (Park et al., 2017; Rheinbay et al., 2013). We performed ChIP‐seq for ASCL1 in the two PDX‐GBMs lines, both of which express high levels of ASCL1 (Figure 1e–m), to identify its target genes in vivo. Using stringent peak calling criteria (Borromeo et al., 2014, 2016), we identified 9,816 statistically significant peaks in the genome of R548‐PDX‐GBM and 7,848 peaks in R738‐PDX‐GBM (blue rectangles, Figure 2a). Although only 4,207 of the significant peaks called overlapped in both PDX‐GBMs, heatmaps of the ASCL1 ChIP‐seq signal intensity, even for the nonsignificant peaks for each PDX‐GBM, was noticeably higher than background for the combined 13,457 peaks called, indicating that the ASCL1 binding profile was similar in both PDX‐GBMs (Figure 2a) (see Table S1 for ASCL1 binding peaks and coordinates).

To validate the quality and efficiency of our ChIP‐seq, we next analyzed ASCL1 binding at known canonical targets (*DLL1*, *DLL3*, *NOTCH1*, *HES5*, *HES6*, and *INSM1*) which have previously been shown to be directly regulated by ASCL1 in numerous contexts (Borromeo et al., 2014, 2016; Castro et al., 2011; Jacob et al., 2009; Park et al., 2017; Somasundaram et al., 2005; Ueno et al., 2012; Vias et al., 2008). As expected, ChIP‐seq tracks revealed the presence of strong ASCL1 binding peaks at loci of all the canonical target genes examined (asterisk, Figure 2b). Moreover, ASCL1 is known to preferentially bind to degenerate CANNTG E‐box motifs to regulate gene expression (Borromeo et al., 2014, 2016; Casey et al., 2018; Castro et al., 2011). Using de novo motif analysis (Heinz et al., 2010), we identified the bHLH CAGCTG E‐box motif as being highly enriched directly beneath 74% of the 13,457 ASCL1 combined peaks called, further confirming the quality of the ChIP‐seq. Interestingly, we found that SOX and FOXO motifs were also significantly enriched within ASCL1 binding peaks (Figure 2c), suggesting that ASCL1 may function in combination with these transcription factor families to regulate gene expression in GBMs.

To identify putative‐targets of ASCL1 in GBMs, we then used GREAT (McLean et al., 2010) to associate nearby genes that were upstream or downstream of the 13,457 ASCL1 binding peaks. From this analysis, we uncovered a total of 8,791 genes (red oval, Figure 2d) (see Table S2 for list of ASCL1 target genes). We reasoned that if these genes are regulated by ASCL1 then they should also be expressed in a manner correlated with *ASCL1* expression in GBMs. By applying Spearman's rank‐ordered correlation (>0.4) to RNA‐seq of the 164 TCGA GBM samples, we then identified the top 10% of genes that showed a positive correlation with *ASCL1* expression across these tumor samples. We found 2,136 genes that are positively correlated with *ASCL1* expression (green oval, Figure 2d) (see Table S3 for list of *ASCL1* correlated genes in GBMs). When we cross referenced these 2,136 genes with the 8,791 genes identified from the ASCL1 ChIP‐seq, there was an overlap of 1,106 genes, which we define as ASCL1 putative target genes in GBM (yellow area, Figure 2d). Supporting the validity of this approach, all ASCL1 canonical targets examined were included in this 1,106 putative‐target gene list (Figure 2d) (see Table S4 for list of ASCL1 putative targets).

By evaluating the ASCL1 putative‐target gene list (Table S4), we uncovered a variety of genes that are particularly relevant to GBM development. Indeed, some of the most notable target genes include other neurodevelopmental and/or glial transcription factors such as OLIG genes (*OLIG1*, *OLIG2*), SOX genes (*SOX1*, *SOX2*, *SOX3*, *SOX4*, *SOX6*, *SOX8*, *SOX10*), NFI genes (*NFIA*, *NFIB*, *NFIX*), POU domain genes (*POU3F2*, *POU3F3*, *POU6F1*), Sal‐like genes (*SALL2*, *SALL3*), and homeobox genes (*NKX2.2*, *ZEB1*). The functions of OLIG2 (Ligon et al., 2007; Lu et al., 2016; Mehta et al., 2011), SOX2 (Gangemi et al., 2009; Singh et al., 2017), and NFIA (Glasgow et al., 2017; Lee, Hoxha, & Song, 2017) have previously been reported to be important for regulating the tumorigenic property of glioma cell lines and in glioma mouse models. Other prominent ASCL1 target genes also include numerous cell cycle (*CCND2*, *CCNE2*, *CDC25C*, *CDK4*, *CDK5R1*, *CSNK1E*, *E2F2*, *MCPH1*, *POLA2*, *PRIM2*), mitosis (*AURKB*, *BRSK1*, *MCM10*, *RCC2*), chromatin modification (*CHD3*, *CHD6*, *CHD7*, *KDM3B*, *KDM4B*, *SMARCA*, *SMARCB1*, *SMARCD1*), as well as oncogenic signal transduction related genes (*AKT3*, *EGFR*, *ERBB3*, *GSK3β*, *MYCN*, *PIK3R1*, *RTKN*, *TCF7L1*, *TCF7L2*). Strong ASCL1 binding peaks at the loci of some of these genes in the PDX‐GBMs lines are illustrated (asterisks, Figure 2e–h).

We next wanted to know how the expression of the 1,106 ASCL1 putative‐target genes sort across the various GBM subtypes using RNA‐seq of the 164 primary GBMs. Heatmap and dendrogram analysis revealed that, similar to *ASCL1*, the 1,106 putative‐target genes were highly expressed in the proneural and classical GBM subtypes, in the majority of neural and mixed GBM subtypes, but was mostly absent in the mesenchymal GBM subtype (Figure 2i). In all, 109 of the TCGA GBM samples were positive for the ASCL1 putative‐target genes, while the remaining 55 samples express very little or low levels of the ASCL1 putative‐targets.

To gain insights into the collective significance of the 1,106 ASCL1 putative targets, we then performed gene set over‐representation analysis to annotate their function using ConsensusPathDB, a comprehensive collection of molecular interaction databases integrated from multiple public repositories (Herwig et al., 2016). Interestingly, the top most enriched pathway identified was cell cycle (Figure 2j). This is consistent with a previous report showing that positive and negative cell cycle regulators in neural progenitor cells are targets of ASCL1 (Castro et al., 2011). Other pathways that are also enriched for ASCL1 targets include those involved in chromatin segregation such as Aurora B Signaling and Amplification of Signal from Kinetochores, and intracellular signaling pathways such as those involved in PIP3 Activates AKT Signaling, Signaling by Rho GTPases, Hippo Signaling Pathway, and Wnt Signaling Pathway & Pluripotency. Finally, cancer pathways such as Retinoblastoma in Cancer, Hepatocellular Carcinoma, and Endometrial Cancer were also enriched for ASCL1 targets (Figure 2j) (see Table S5 for list ASCL1 target enriched biological pathways). Taken together, these findings suggest that ASCL1 is a transcriptional regulator at the epicenter of multiple biological processes that are fundamental to cancer development.

### 3.3 ASCL1, OLIG2, and SOX2 are coexpressed in early and terminal stage tumors of a glioma mouse model

**FIGURE 3:**
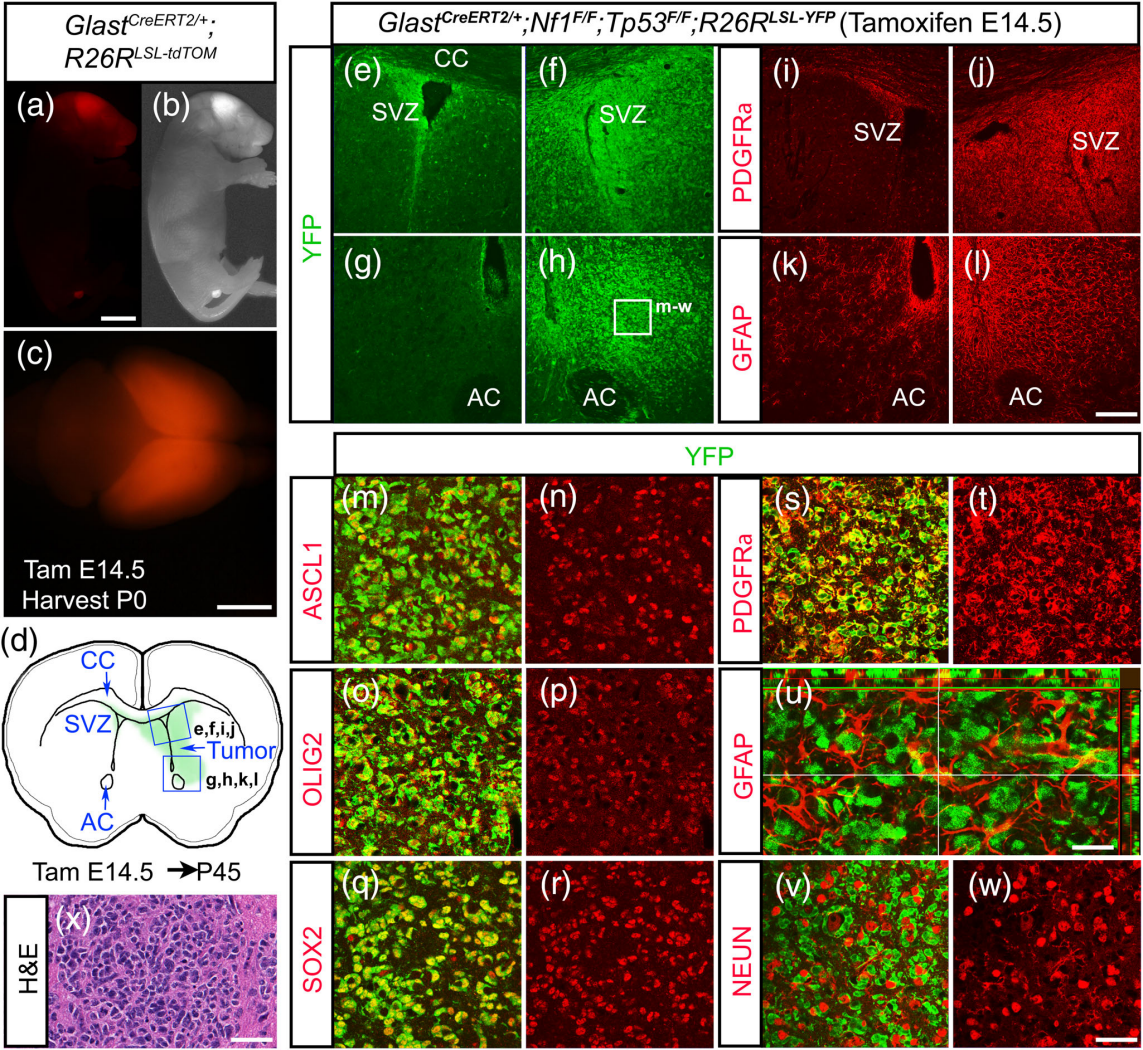
ASCL1, OLIG2, and SOX2 are highly expressed in early stage tumor cells of the glioma mouse model. (a–c) A neonatal pup from *Glast* ^*CreERT2/+*^ crossed with *R26R* ^*LSL‐tdTomato*^ reporter administered with tamoxifen at E14.5. Note that tdTomato fluorescence is specific to the CNS and highest in the cerebral cortex (a and c). (d) Schematic of an early stage brain tumor surrounding the right subventricular zone (SVZ) of a *Glast* ^*CreERT2/+*^ *;Nf1* ^*F/F*^ *;* ^*F/F*^ *;R26R* ^*LSL‐YFP*^ mouse, administered with tamoxifen at E14.5 and harvested at P45. (e–l) Immunofluorescence shows high YFP reporter expression (e–h), OPC marker PDGFRα (i and j) and astrocyte marker GFAP (k and l) in tumor areas indicated in (d). (m–w) Higher magnification of tumor area indicated in (h) showing ASCL1 (m and n), OLIG2 (o and p), SOX2 (q and r), and PDGFRα (s and t) colocalized with YFP in tumor cells, but not GFAP (u) or the neuronal marker NEUN (v, w). (x) H&E staining of an early stage tumor exhibiting characteristic feature of glioma. Scale bar is 5 mm for (a and b); 3 mm for (c); 100 μm for (e–l); 25 μm for (m–t, v–x); and 12.5 μm for (u) [Color figure can be viewed at wileyonlinelibrary.com]

To functionally test ASCL1's role in gliomagenesis in vivo, we began by characterizing the temporal expression pattern of ASCL1 along with OLIG2, SOX2, and glial lineage markers in brain tumors induced in a mouse model carrying floxed alleles of the tumor suppressor genes Neurofibromin 1 (*Nf1*) and tumor protein 53 (*Tp53*) (*Nf1* ^*F/F*^ *;Tp53* ^*F/F*^) (Lin et al., 2004; Zhu et al., 2001). *NF1* and *TP53* are two of the most highly mutated genes in human GBM (Brennan et al., 2013; Verhaak et al., 2010), and Cre‐recombinase deletion of these two tumor suppressor genes (*Nf1;Tp53* ^*CKO*^) in neural progenitors or glial precursor cells have previously been shown to be fully penetrant in producing glioma tumors in the brains of mice (Alcantara Llaguno et al., 2009; Alcantara Llaguno et al., 2015; Zhu et al., 2005). When mice carrying a *Glast* ^*CreERT2/+*^ knock‐in allele (Mori et al., 2006) was crossed with the *Rosa26‐loxP‐stop‐loxP‐tdTomato* (*R26R* ^*LSL‐tdTom*^) reporter line (Madisen et al., 2010), we found that tdTomato fluorescence was restricted in the brain of neonatal pups if tamoxifen was administered at E14.5 (Figure 3a–c), making *Glast* ^*CreERT2/+*^ ideal to combine with the *Nf1* ^*F/F*^ *;Tp53* ^*F/F*^ alleles to induce brain tumors.

To visualize the tumors as they develop in the brain, a *R26R* ^*LSL‐YFP*^ reporter allele (Srinivas et al., 2001) was incorporated into the glioma mouse model (*Glast* ^*CreERT/+*^ *;Nf1* ^*F/F*^ *;Tp53* ^*F/F*^ *;R26R* ^*LSL‐YFP*^). Tamoxifen was then administered to pregnant dams at E14.5 to induce *Nf1;Tp53* ^*CKO*^ in neural progenitors of embryos. We first analyzed early tumors in the offspring at postnatal day (P) 45, at which point the majority of the mice were still asymptomatic and have yet to exhibit neurological symptoms. As expected, we were able to observe the presence of a tumor in some mice marked by intense YFP expression typically on one side of the brain surrounding the ventricle (Figure 3d–h). The tumor at this stage was not easily distinguishable from nontumor tissues without YFP immunohistochemistry, yet both PDGFRα, an oligodendrocyte precursor cell (OPC) marker, and GFAP, an astrocyte marker, were ectopically expressed on the tumor side, indicating that the tumor is a glioma (Figure 3i–l). High magnifications revealed that ASCL1, OLIG2, and SOX2 are also expressed specifically within the YFP+ tumor cells (Figure 3m–r), which are highly irregular in shape, morphology, and density compared to normal YFP+ cells on the nontumor side (not shown). H/E staining also confirmed that these early tumors exhibited histological characteristics of gliomas (Figure 3x). Interestingly, the YFP+ tumor cells colocalized extensively with PDGFRα (Figure 3s,t) but not with GFAP or the neuronal marker NEUN (Figure 3u–w). The lack of colocalization between YFP and GFAP was similar to that observed in tumors of another glioma mouse model in which PDGF stimulation was combined with deletion of another tumor suppressor, *Pten* (Lei et al., 2011), and implies that the majority of GFAP+ cells are reactive astrocytes that have infiltrated the YFP+ tumor bulk in our model.

**FIGURE 4:**
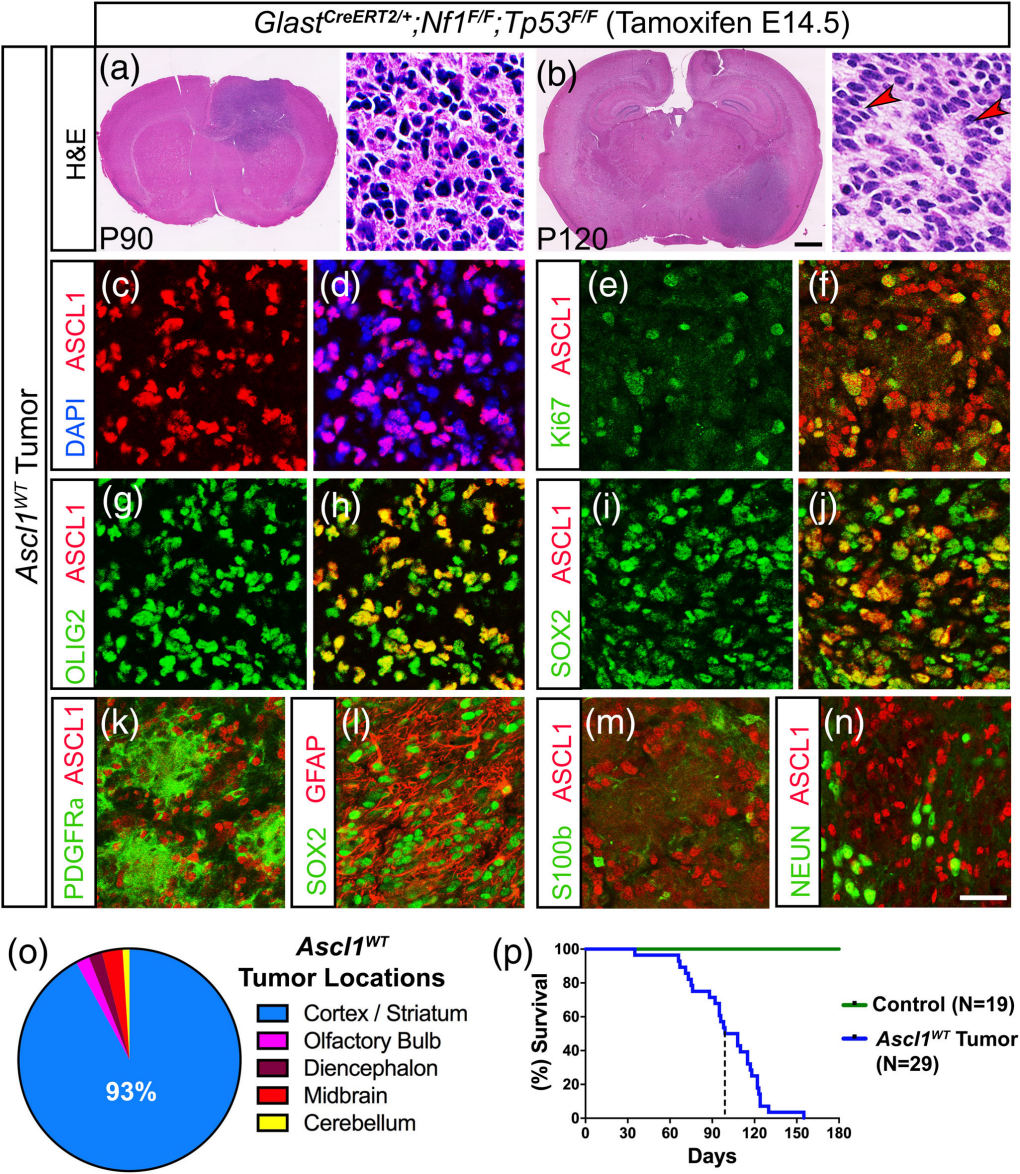
Expression of ASCL1, OLIG2, and SOX2 are maintained in mice with terminal stage glioma tumors. (a and b) H&E staining of *Ascl1* ^*WT*^ terminal stage tumors harvested at P90 and P120. Higher magnification insets show that tumors are high‐grade gliomas. Arrowheads indicate pseudopalisading cellular features consistent with GBM. (c–n) Immunofluorescence of *Ascl1* ^*WT*^ tumor tissue. ASCL1 is present in the majority of DAPI+ tumor cells (c and d) and colocalizes with Ki67 (e and f), OLIG2 (g and h), and SOX2 (I and j). PDGFRα (k) and GFAP (l) are also coexpressed in ASCL1+ or SOX2+ tumor cells respectively, but not S100β (m) and NEUN (n). (o) Incidence of tumors observed in different brain regions is indicated. Over 90% of tumors are found in the cortex and striatum (*N* = 29). (p) Survival curve of *Ascl1* ^*WT*^ tumor (*N* = 29) bearing mice and Cre‐negative control mice (*N* = 19). Dotted line indicates median survival of 102 days for *Ascl1* ^*WT*^ tumor mice. Scale bar is 1 mm for whole brain section and 30 μm for insets in (a and b); and 25 μm for (c–n) [Color figure can be viewed at wileyonlinelibrary.com]

From P60‐120, we found that 100% of *Nf1;Tp53* ^*CKO*^ mice exhibited neurological symptoms and had tumors that evolved into an expanded mass with high mitotic index and microvascular proliferation resembling that of high‐grade gliomas (Figure 4a,b). We termed these *Ascl1* ^*WT*^ tumor mice (*N* = 29, blue line), which exhibited a median survival of 102 days, while CreER‐negative littermate controls (*N* = 19, green line) were tumor‐free and healthy (Figure 4p). Over 90% of the tumors were found in the cortex and/or striatum area, while a minority was also found in olfactory bulb, diencephalon, midbrain, or cerebellum (Figure 4o). Similar to the early tumors and the PDX‐GBMs, ASCL1, OLIG2, and SOX2 were coexpressed in the tumor cells of these terminal tumors (Figure 4c,d,g–j), and many ASCL1+ tumor cells were also Ki67+ (Figure 4e,f). PDGFRα was also highly coexpressed by the ASCL1+ (Figure 4k) and OLIG2+ (not shown) tumor cells, whereas GFAP and to a lesser extent S100β and NEUN, although found in some parts of the tumor, did not overlap significantly with SOX2 or ASCL1 (Figure 4l–n).

Overall, our findings illustrate that ASCL1, OLIG2, and SOX2 are coexpressed in tumor cells of both early and terminal tumors of the glioma mouse model in vivo, and tumor cells maintain a molecular identity reminiscent of that of OPCs.

### 3.4 Loss of ASCL1 decreases the proliferation of gliomas and increases the survival of tumor bearing mice

Currently, the direct requirement of ASCL1 in brain tumor formation and progression from low‐grade gliomas to high‐grade GBMs in vivo remains unknown. To address this, we incorporated *Ascl1* ^*GFP*^ knock‐in (null) and *Ascl1* ^*Floxed*^ alleles into the glioma mouse model to generate *Glast* ^*CreERT2*^ *;Ascl1* ^*GFP/F*^ *;Nf1* ^*F/F*^ *;Tp53* ^*F/F*^ and *Glast* ^*CreERT2*^ *;Ascl1* ^*F/F*^ *;Nf1* ^*F/F*^ *;Tp53* ^*F/F*^ mice, respectively, both of which when administered with tamoxifen at E14.5 will result in triple conditional knock‐out of *Ascl1* along with *Nf1* and *Tp53* (*Ascl1;Nf1;Tp53* ^*CKO*^). To control for the possible effects of genetic background on glioma phenotype observed, we also generated *Glast* ^*CreERT2*^ *;Ascl1* ^*GFP/+*^ *;Nf1* ^*F/F*^ *;Tp53* ^*F/F*^ and *Glast* ^*CreERT2*^ *;Ascl1* ^*F/+*^ *;Nf1* ^*F/F*^ *;Tp53* ^*F/F*^ mice in parallel for comparison, both of which developed tumors that are still heterozygous for *Ascl1* when induced with tamoxifen, and are referred to as *Ascl1* ^*HET*^ tumor mice.

**FIGURE 5:**
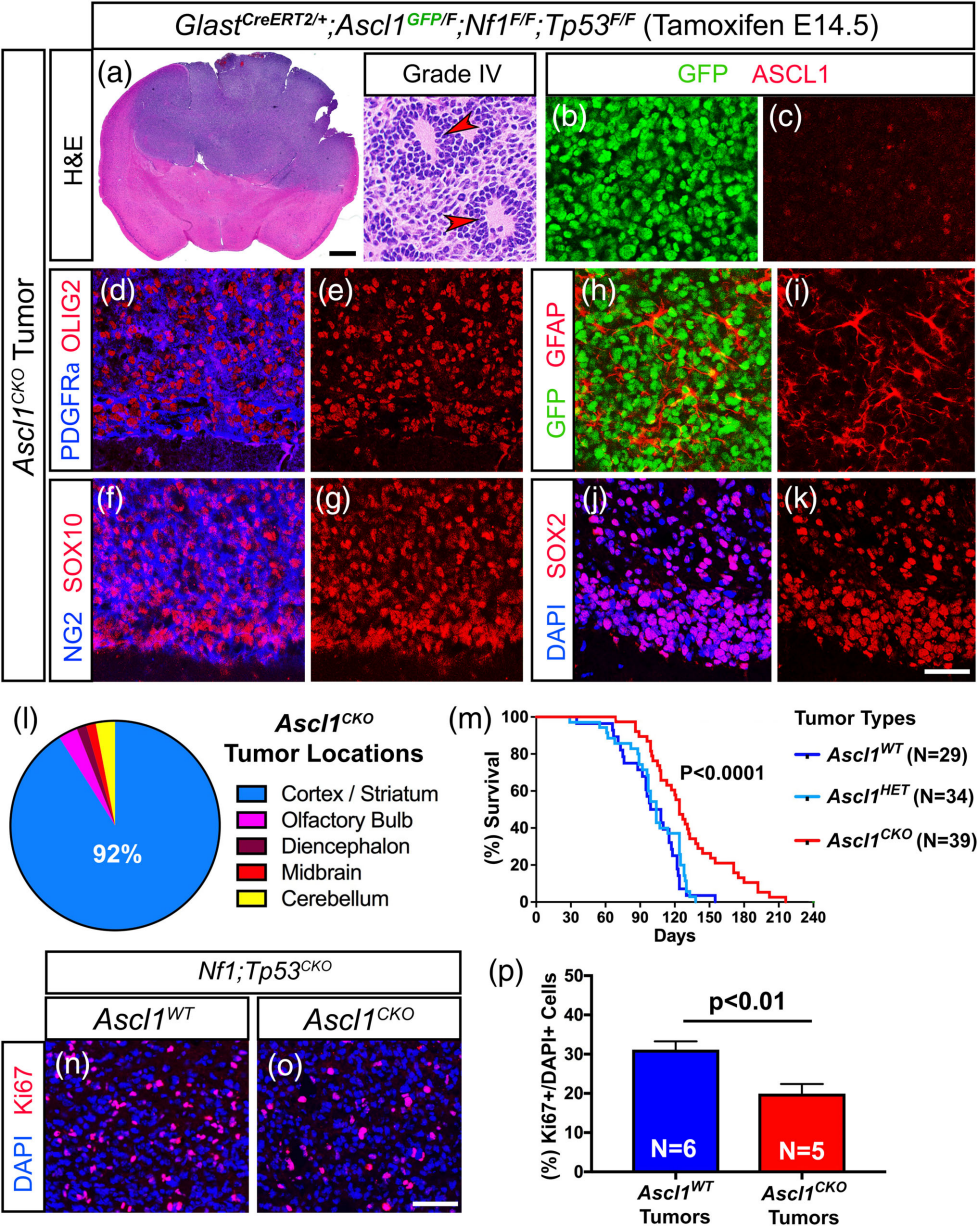
Survival of glioma tumor bearing mice is increased in the absence of ASCL1. (a) H&E staining of an *Ascl1* ^*CKO*^ tumor exhibiting pseudopalisading cellular features of Grade IV glioma (arrowheads, insets). (b–k) Immunofluorescence of *Ascl1* ^*CKO*^ tumor. GFP, driven by an *Ascl1* ^*GFP*^ knock‐in allele, is present in tumor cells but ASCL1 is absent (b and c), indicating efficient deletion of *Ascl1* ^*Floxed*^ allele. Expression of OLIG2 and PDGFRα (d and e), SOX10 and NG2 (f and g), GFAP (h and i), and SOX2 (j and k) are unaffected. (l) Incidence of *Ascl1* ^*CKO*^ tumors observed in the different brain regions. Over 90% of tumors are found in the cortex and striatum area similar to *Ascl1* ^*WT*^ tumors. (m) Survival curve of *Ascl1* ^*CKO*^ versus *Ascl1* ^*HET*^ tumor mice. Median survival is significantly improved for *Ascl1* ^*CKO*^ (122 days) compared to *Ascl1* ^*HET*^ (104 days) tumor mice (dotted lines). Note that survival of *Ascl1* ^*HET*^ is very similar to *Ascl1* ^*WT*^ tumor mice (note blue line is the same as Figure 4p). (n–p) Immunofluorescence (n and o) and quantification of the percentage of Ki67+/DAPI+ tumor cells (p) for *Ascl1* ^*WT*^ and *Ascl1* ^*CKO*^ tumors. Scale bar is 1 mm for whole brain section and 30 μm for insets in (a); 25 μm for (b–k); and 50 μm for (n and o) [Color figure can be viewed at wileyonlinelibrary.com]

Previous reports demonstrate that ASCL1 is essential for the proliferation of GBM cell lines in vitro (Park et al., 2017; Rheinbay et al., 2013). In contrast, in vivo we found that *Ascl1;Nf1;Tp53* ^*CKO*^ mice (hence forth referred to as *Ascl1* ^*CKO*^ tumor mice, *N* = 39) still developed high‐grade tumors that were phenotypically consistent with high‐grade gliomas (Figure 5a). Furthermore, *Ascl1* ^*CKO*^ tumor penetrance and location (Figure 5l,m) in the brain was similar to the *Ascl1* ^*HET*^ (not shown) and *Ascl1* ^*WT*^ tumors (Figure 4o,p). We confirmed that ASCL1 was indeed absent in *Ascl1* ^*CKO*^ tumors. As illustrated for a *Glast* ^*CreERT2*^ *;Ascl1* ^*GFP/F*^ *;Nf1* ^*F/F*^ *;Tp53* ^*F/F*^ mouse, GFP driven by the endogenous *Ascl1* locus marks precisely the tumor cells but ASCL1 was no longer detected (Figure 5b,c). Notably, OLIG2 and SOX2 (Figure 5d,e,j,k), which we identified as ASCL1 target genes, were still expressed, indicating that expression of these two transcription factors do not depend solely on ASCL1. Similarly, OPC markers such as PDGFRα, the chondroitin sulfate NG2, and SOX10 were still expressed in the *Ascl1* ^*CKO*^ tumors (Figure 5d,f,g). As observed in *Ascl1* ^*WT*^ tumors, GFAP did not cocolocalize extensively with GFP+ tumor cells, despite being expressed in some regions of the tumor (Figure 5h,i). Together, these findings demonstrate that glial transcription factors and the OPC‐like identity of the tumor cells are still retained in the absence of ASCL1.

Notably, the *Ascl1* ^*CKO*^ tumor mice survived longer compared to *Ascl1* ^*HET*^ and *Ascl1* ^*WT*^ tumor mice. Specifically, while the *Ascl1* ^*HET*^ tumor mice (*N* = 34) died between P60‐130, with a median survival of 104 days, which is very similar to *Ascl1* ^*WT*^ tumor mice (median survival of 102 days), *Ascl1* ^*CKO*^ tumor mice (*N* = 39) died later between P90‐180, with a median survival of around 122 days (compare red vs. light and dark blue lines, Figure 5m). This improvement in survival for the *Ascl1* ^*CKO*^ tumor mice also holds true even when analyzed by gender (not shown) and strongly suggests that it was due to the loss of ASCL1.

To determine what may account for the improved survival of the *Ascl1* ^*CKO*^ tumor mice, we assessed tumor proliferation by quantifying the percentage of tumor cells that were Ki67+ in comparison to *Ascl1* ^*WT*^ tumor mice. Because the density of Ki67+ cells can vary dramatically across a large tumor depending on necrosis or the integrity/quality of the tumor tissue, we chose to image and quantify several regions of each *Ascl1* ^*CKO*^ (*N* = 5) or *Ascl1* ^*WT*^ tumor (*N* = 6) with the highest density of Ki67+ cells (Figure 5n,o). Overall, *Ascl1* ^*CKO*^ tumors exhibited a decrease of about 30% Ki67+ cells compared to *Ascl1* ^*WT*^ tumors (Figure 5p), which is consistent with our previous finding that numerous cell cycle and mitotic genes are targets of ASCL1. This decrease in Ki67+ cells was similar to that observed for adult OPCs in the spinal cord when *Ascl1* was conditionally deleted (Kelenis, Hart, Edwards‐Fligner, Johnson, & Vue, 2018) and supports the interpretation that the increased survival of *Ascl1* ^*CKO*^ tumor mice may result from a decrease in the rate of tumor cell proliferation.

### 3.5 Transcriptome of mouse GBM tumors showed that loss of ASCL1 is associated with downregulation of cell cycle genes

**FIGURE 6:**
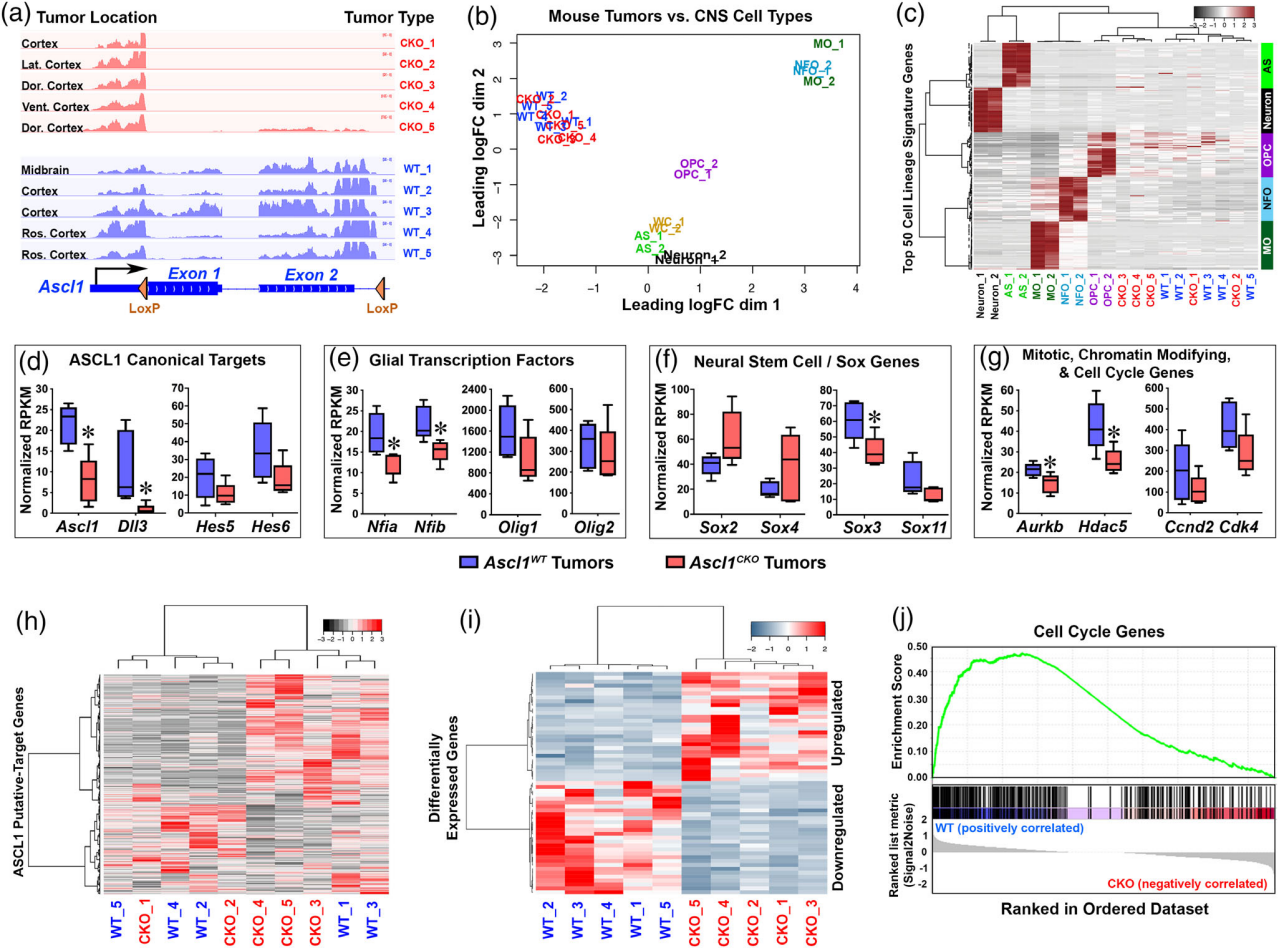
Cell cycle genes are downregulated in *Ascl1* ^*CKO*^ glioma tumors of the mouse model. (a) RNA‐seq tracks at the *Ascl1* locus of *Ascl1* ^*WT*^ and *Ascl1* ^*CKO*^ tumors isolated from brain regions indicated. Note that Exon 1 and 2 of the *Ascl1 mRNA*, flanked by Lox P sites, are absent in *Ascl1* ^*CKO*^ tumors. (b) Multidimensional scaling (MDS) plot of RNA‐seq of *Ascl1* ^*WT*^ and *Ascl1* ^*CKO*^ tumors versus CNS cell types (Zhang et al., 2014). *Ascl1* ^*WT*^ and *Ascl1* ^*CKO*^ tumors are more similar to each other than to any of the CNS cell types. AS, astrocytes; OPC, oligodendrocyte precursor cells; NFO, newly formed oligodendrocytes; MO, myelinating oligodendrocytes; WC, whole cortex. See Table S6 for normalized gene expression (RPKM) in *Ascl1* ^*WT*^ and *Ascl1* ^*CKO*^ tumors. (c) Heatmap and dendrograms using the top 50 CNS cell lineage signature genes for each cell type (Zhang et al., 2014). Dendrograms on top show that *Ascl1* ^*WT*^ and *Ascl1* ^*CKO*^ tumors express signature genes that are more similar to OPCs than to the other CNS cell types. (d–g) Box and whisker plots of ASCL1 putative‐target genes in *Ascl1* ^*WT*^ and *Ascl1* ^*CKO*^ tumors. Canonical targets of ASCL1 (d), glial transcription factors (e), and mitotic, chromatin modifying, and cell cycle genes (g) are expressed at lower level while neural stem cell/*Sox* genes (f) are bidirectionally affected in *Ascl1* ^*CKO*^ compared to *Ascl1* ^*WT*^ tumors. Asterisks indicate target genes significantly altered (*p* < .05, Wilcox test). (h–i) Heatmap and dendrograms of differentially expressed genes (DEGs) in *Ascl1* ^*WT*^ and *Ascl1* ^*CKO*^ GBMs. DEGs consist of ASCL1 putative direct targets (h) (see Table S7) and indirect target DEGs (FDR < 0.05) (i) (see Table S8). (j) Gene‐set‐enrichment‐analysis (GSEA) showing that cell cycle genes are enriched in the downregulated genes in *Ascl1* ^*CKO*^ compared to *Ascl1* ^*WT*^ tumors [Color figure can be viewed at wileyonlinelibrary.com]

To determine if the loss of ASCL1 altered the molecular profiles of the mouse glioma tumors, we carefully isolated tumor bulk from various regions of the brain from *Ascl1* ^*WT*^ (*N* = 5) and *Ascl1* ^*CKO*^ (*N* = 5) tumor mice for RNA‐seq analysis. RNA‐seq tracks of the *Ascl1* locus show that *Exons 1* and *2* of the *Ascl1 mRNA* (containing the entire coding sequence), were completely absent in all *Ascl1* ^*CKO*^ tumors but were present in *Ascl1* ^*WT*^ tumors (Figure 6a), confirming efficient deletion of the *Ascl1* ^*Floxed*^ allele. We first compared the transcriptomes of the *Ascl1* ^*WT*^ and *Ascl1* ^*CKO*^ tumors (Table S6) with transcriptomes of CNS cell types, including OPCs, newly formed oligodendrocytes (NFO), mature oligodendrocytes (MO), astrocytes (AS), neurons, and whole cortex (WC) (Zhang et al., 2014). A multidimensional scaling (MDS) plot shows that both the *Ascl1* ^*WT*^ and *Ascl1* ^*CKO*^ tumors cluster together and away from the CNS cell types, and therefore are more similar to each other than to neurons or any of the glial lineage cells (Figure 6b). When we further analyzed RNA‐seq of *Ascl1* ^*WT*^ and *Ascl1* ^*CKO*^ tumors using the top 50 signature genes for each CNS cell type, both tumor types more closely resemble that of OPCs rather than the other CNS cell types (Figure 6c). This finding further supports the notion that OPCs, which are highly proliferative and migratory, may be the precursor cell‐of‐origin for the glioma tumors in this model.

Finally, we sought to identify genes that are differentially expressed between *Ascl1* ^*WT*^ and *Ascl1* ^*CKO*^ tumors. Analysis of ASCL1 canonical target genes revealed that *Dll3*, similar to *Ascl1*, was significantly decreased, while *Hes5* and *Hes6* were lowered (Figure 6d), but *Dll1*, *Notch1*, and *Insm1* (not shown) were unchanged in *Ascl1* ^*CKO*^ tumors. Interestingly, glial transcription factors *Nfia* and *Nfib*, and several mitotic (*Aurkb*) and chromatin modifying (*Hdac5*) genes were significantly decreased, whereas *Olig1*, *Olig2*, and cell cycle genes (*Ccnd2* and *Cdk4*) were somewhat reduced in *Ascl1* ^*CKO*^ tumors (Figure 6e,g). In contrast, *Sox* genes were bidirectionally affected by the loss of ASCL1. For instance, although *Sox3* and *Sox11* were decreased, *Sox2* and *Sox4* appeared upregulated in the *Ascl1* ^*CKO*^ tumors (Figure 6f). Heatmap and dendrogram analysis of all 1,054 ASCL1 putative targets (converted from a list of 1,106 genes from human GBMs, Table S4), revealed that there were as many genes being upregulated as there were genes being downregulated by the loss of ASCL1 (Figure 6h, Table S7). We also identified about 50 indirect targets of ASCL1 that were either upregulated or downregulated in the *Ascl1* ^*CKO*^ tumors (Figure 6i, Table S8). Finally, in agreement with our earlier finding that tumor cell proliferation is decreased in the absence of ASCL1, gene‐set‐enrichment analysis revealed that cell cycle related genes were highly enriched in the downregulated genes in the *Ascl1* ^*CKO*^ tumors (Figure 6j). This suggests that a decreased in cell‐cycle related gene expression may contribute to the increase in survival of the *Ascl1* ^*CKO*^ tumor mice.

In summary, our findings highlight an in vivo role for ASCL1 in modulating the expression of a variety of genes, including neurodevelopmental or glial transcription factors and cell cycle genes, either directly or indirectly, that are crucial for the proliferation of glioma tumors in the brains of genetically relevant mouse models.

## 4 DISCUSSION

We demonstrate in this study that ASCL1 is highly expressed in the majority of PDX‐GBM cells in vivo, with over 90% of ASCL1+ cells coexpressing OLIG2 and SOX2. Interestingly, in addition to OLIG2 and SOX2, we find that expression of a variety of other genes encoding transcription factors such as those for NFI, POU domain, Sal‐like, SOX, and homeobox families are also highly correlated with *ASCL1* expression in RNA‐seq of primary GBMs (Table S3). This finding is similar to that previously reported in GSCs from cultured GBM cell lines (Rheinbay et al., 2013; Suva et al., 2014). Accordingly, we find that these transcription factor encoding genes are also targets of ASCL1 binding (Tables S1 and S2). These findings support a complex transcription factor interaction network in which the coexpression of these transcription factors may be interdependent on each other, and this coexpression is essential for regulating genes crucial for maintaining glioma cells in an aberrant stem‐like state of dedifferentiation and proliferation. In agreement with this, it is not surprising that combinatorial overexpression of multiple transcription factors is necessary and sufficient to reprogram differentiated glioma cells or immortalized astrocytes into tumor propagating cells (Singh et al., 2017; Suva et al., 2014).

ChIP‐seq for ASCL1 has previously been performed for glioma cell lines in culture revealing that ASCL1 directly interacts with Wnt signaling by binding to genes such as *AXIN2*, *DKK1*, *FZD5*, *LGR5*, *LRP5*, *TCF7*, and *TCF7L1*. A model was proposed in which ASCL1 functions at least in part by repressing an inhibitor of Wnt signaling, *DKK1*, resulting in increased signaling through this pathway to maintain the tumorigenicity of glioma cells (Rheinbay et al., 2013). Analysis of microarray data in primary GBM cultures also divided GSCs into ASCL1^high^ and ASCL1^low^ subgroups, where the former is closely correlated with the proneural subtype, whereas the latter is associated with the mesenchymal subtype (Park et al., 2017). Notably, dual attenuation of both Wnt and Notch signaling in the ASCL1^high^ subgroup upregulated a neurogenic program similar to that observed during development, resulting in decreased tumorigenicity of GSCs (Park et al., 2017; Rajakulendran et al., 2019). Together, these studies support the conclusion that ASCL1 levels in gliomas acts in a balance with both Wnt and Notch signaling pathways to regulate the GSC status of tumor cells in culture.

Here we also find that both Wnt and Notch related genes are also directly bound by ASCL1 in PDX‐GBMs grown orthotopically in the brains of mice. Additionally, expression of many of these genes is positively correlated with *ASCL1* expression when analyzed across RNA‐seq of the 164 TCGA primary GBM samples, and Wnt and Notch Signaling were identified as some of the pathways significantly over‐represented by the ASCL1 target genes that we identified in the PDX‐GBMs (Figure 2j, Table S2). Despite these findings, RNA‐seq from a *Nf1;Tp53* ^*CKO*^ mouse model of glioma where the tumors also lack ASCL1 revealed that although some Notch related genes (*Dll3*, *Hes5*, *Hes6*) were decreased, expression of many of the Wnt related genes seemed unaffected by the loss of ASCL1. Thus, although ASCL1 binds to and may contribute to the regulation of some of these target genes, particularly in an in vitro setting (Rheinbay et al., 2013), expression of many of these target genes remains in gliomas in vivo in the absence of ASCL1.

Interestingly, ChIP‐seq for ASCL1 in PDX‐GBMs and RNA‐seq of mouse *Ascl1* ^*CKO*^ tumors in our study revealed that cell cycle and mitotic genes are major targets of ASCL1 binding (Figure 2f,j), and these targets were most impacted by the loss of ASCL1 (Figure 6g,j). In agreement with this, the percentage of Ki67+ cells was significantly decreased in *Ascl1* ^*CKO*^ relative to *Ascl1* ^*WT*^ tumors of the mouse model (Figure 5n–p). This decrease in proliferation may have led to the increased survival time for mice with tumors lacking ASCL1. Taken together, these findings demonstrate that a major role for ASCL1 in the *Nf1;Tp53* ^*CKO*^ glioma mouse model is to drive tumor cell proliferation. This function of ASCL1 is similar to what has been observed in neural progenitor cells during development as well as in the adult brain. In particular, although overexpression of ASCL1 is predominantly known to promote cell cycle exit, cell fate specification, and neuronal differentiation, a prominent phenotype associated with *Ascl1* mutants is a loss in the overall progenitor pool as a result of a decrease in proliferation and a disruption in the ASCL1/NOTCH balance, which is essential to maintain the progenitor pool (Borromeo et al., 2014; Casarosa, Fode, & Guillemot, 1999; Castro et al., 2011; Horton, Meredith, Richardson, & Johnson, 1999; Nakada, Hunsaker, Henke, & Johnson, 2004). As a validation of ASCL1's direct role in progenitor cell proliferation, ChIP‐seq of mouse embryonic telencephalon, adult neural progenitors, and neural stem cells in cultures also found that ASCL1 directly binds to a large number of genes involved in cell cycle progression (Andersen et al., 2014; Castro et al., 2011). These target genes include positive cell cycle regulators and oncogenic transcription factors, some of which are also identified here in our ChIP‐seq of ASCL1 in the PDX‐GBMs (Table S2). Similarly, within regions of adult neurogenesis such as the subventricular zone (SVZ) of the lateral ventricle and subgranular zone (SGZ) of the hippocampal dentate gyrus, ASCL1 is expressed at very low level or is undetectable in quiescent neural stem cells which exhibit radial glial‐like morphology and express GFAP and Nestin. In contrast, ASCL1 expression is highest in transiently amplifying progenitors (TAPs), which are highly proliferative (Kim et al., 2011). Conditional knock‐out studies have shown that ASCL1 is responsible for promoting neural stem cells from a quiescent into an activated state in the SGZ, and the number of proliferating progenitors is significantly decreased with the loss of ASCL1 (Andersen et al., 2014; Kim et al., 2011; Urbán et al., 2016). Using a similar conditional knock‐out approach, we also demonstrated that ASCL1 is specifically required for the proliferation of OPCs in both embryonic and adult spinal cord (Kelenis et al., 2018). Indeed, the level of ASCL1 is positively correlated with the rate of OPC proliferation (Kelenis et al., 2018; Nakatani et al., 2013), and the decrease in proliferation of OPCs in the absence of ASCL1 is similar to that observed for *Ascl1* ^*CKO*^ tumors.

In addition to gliomas, ASCL1 is highly expressed in cancers with neuroendocrine characteristics from multiple tissues including SCLC, prostate cancer, and thyroid medullary carcinoma (Chen, Kunnimalaiyaan, & Van Gompel, 2005; Rapa et al., 2013; Zhang et al., 2018). Previously, we reported that ASCL1 is required for tumor formation in a mouse model of SCLC (Borromeo et al., 2016). This finding reflects the requirement for ASCL1 in the generation and survival of pulmonary neuroendocrine cells (PNECs), a presumptive cell‐of‐origin for SCLC. In contrast, we find that ASCL1 is not required for glioma formation in the brain of the mouse model, although disease progression is altered and the animals have extended survival. Based on cell lineage markers in the glioma mouse model used here, our finding implicates OPCs as the presumptive cell‐of‐origin for the tumors. OPC specification and generation in the CNS is dependent upon OLIG2 (Lu et al., 2002; Zhou, Choi, & Anderson, 2001); however, ASCL1 also plays an important role to regulate the number and proliferation of OPCs (Kelenis et al., 2018; Nakatani et al., 2013; Parras et al., 2007; Vue et al., 2014). Interestingly, in addition to ASCL1 and OLIG2, transcription factors such as NFIA, SOX2, and SOX10 are also expressed in OPCs. However, as OPCs differentiate to become mature oligodendrocytes, only OLIG2 and SOX10 are maintained while ASCL1, NFIA, and SOX2 are downregulated (Glasgow et al., 2014; Laug, Glasgow, & Deneen, 2018; Nakatani et al., 2013). This downregulation suggests that the coexpression of these transcription factors is important for maintaining OPCs in a progenitor‐like state, and the loss of just one of these factors does not completely abrogate tumor formation following deletion of *Nf1* and *Tp53* because OPCs are still generated, and are thus susceptible to being transformed into glioma. It is also possible that within the context of cancer, mutations to tumor suppressor and/or oncogenes may result in dysregulation of ASCL1 and the other transcription factors to be aberrantly coexpressed in neural progenitors or OPCs, thereby suppressing their differentiation and maintaining these cells in a constant state of proliferation, eventually leading to glioma formation. Recently, the direct roles of NFIA and OLIG2 in tumor development in glioma mouse models were also tested. Similar to our findings for ASCL1, tumor formation persisted in the absence of each of these transcription factors. Furthermore, despite utilizing different approaches and driver mutations to induce tumor formation, the loss of NFIA or OLIG2 was also accompanied by significant decreases in tumor cell proliferation resulting in an increase in survival for their respective mouse models (Glasgow et al., 2017; Lu et al., 2016). Together, these studies illustrate potential redundant roles for neurodevelopmental or glial transcription factors in driving glioma formation and progression in vivo in the brain, where the loss of one factor does not completely prevent tumor formation and progression possibly because of compensation by the remaining transcription factors.

Similar to the findings reported here, deletion of *Nf1*, *Tp53*, along with or without *Pten* at adult stages, produces glioma tumors in the brains of mice using multiple neural stem/progenitor cell type specific Cre drivers (Alcantara Llaguno et al., 2009; Alcantara Llaguno et al., 2015; Zhu et al., 2005). Tumors were induced from neural progenitors in the SVZ or OPCs, leading to the formation of two types of glioma tumors (Alcantara Llaguno et al., 2015). Type 1 tumors were found in dorsal/anterior brain regions such as striatum, hippocampus, and cortex, are highly infiltrative and aggressive, and express high levels of GFAP. Type 2 tumors, on the other hand, were found more in ventral/posterior brain regions such as the diencephalon and brainstem, exhibit well‐defined boundaries, and express high levels of OLIG2 and PDGFRα. Based on difference in gene expression, Type 1 tumors are speculated to be derived from neural stem/progenitor cells in the SVZ, whereas Type 2 tumors are likely to be derived from OPCs. In the glioma model used here, in which Cre is driven in neural progenitors at embryonic stages, over 90% of the mouse glioma tumors were found in the telencephalon, predominantly in the cortex and striatum, suggesting that they may be similar to Type 1 tumors. These tumors express high levels of GFAP, OLIG2, SOX10, NG2, and PDGFRα, although we show that the majority of GFAP expressing cells do not colocalize with the Cre‐reporter, which directly marks the oncogenic cells. Whether this is also true for the Type 1 tumors described is not known since direct colocalization with GFAP was not assessed.

In conclusion, the tumors induced embryonically through deletion of *Nf1;Tp53* deletion are highly heterogenous based on RNA‐seq analysis, which is similar to that seen for human GBMs (Patel et al., 2014; Sottoriva et al., 2013). This intertumor heterogeneity is likely the result of different tumors being spontaneously derived from different cell‐of‐origins in the various brain regions, and are thus exposed to different microenvironments such as microglia, reactive astrocytes, and immune cells, which are known to infiltrate tumor bulk and are included in the RNA‐seq. Despite this heterogeneity, however, the loss of ASCL1 still significantly delays tumor progression and resulted in a significant increase in survival for mice with *Ascl1* ^*CKO*^ tumors over those mice with *Ascl1* ^*WT*^ tumors, illustrating an important role for ASCL1 in controlling the rate of glioma proliferation in vivo. A fundamental question remaining for future studies is whether ASCL1 and other transcription factors involved in tumor formation are similarly required for maintenance of glioma growth in the brain, and how much these transcription factors may contribute to glioma recurrence, if any, following multimodal treatments.

## CONFLICT OF INTEREST

The authors declare no potential conflict of interest.

## AUTHOR CONTRIBUTIONS

Tou Yia Vue performed experiments with assistance from Mark D. Borromeo and Tyler Smith and prepared all the figures; Rahul K. Kollipara performed bioinformatic and statistical analyses; Dennis K. Burns performed histological analysis of brain tumors; Tomoyuki Mashimo and Robert M. Bachoo generated and provided PDX‐GBM mice; Tou Yia Vue and Jane E. Johnson designed the study, analyzed all data, and wrote the manuscript. All authors provided scientific insights and edited the manuscript.

## Supporting information


**Figure S1** Subtype identities of primary GBMs using RNA‐seq. RNA‐seq data of 164 TCGA Primary GBMs and 5 normal brain samples (Brennan et al., 2013). Heatmap and dendrogram using the 840 GBM Subtype Signature Genes (Verhaak et al., 2010) reveals the presence (rectangles) of four previously identified GBM subtypes (PN—proneural, MS—mesenchymal, CL—classical, NE—neural) as well as Mixed GBM group which express multiple subtype signatures.

**Table S1** Contains the ASCL1 ChIP‐seq binding site coordinates in R738 and R548 GBM HOT samples. Peak coordinates are in hg19 genome build. Peaks were called using Homer tool. Peaks from the two samples were compared and listed as shared peaks if the peak coordinates overlapped within 150 bp.

**Table S2** Contains list of genes and associated peak IDs were listed in this table. Union set of peak coordinates were obtained from two samples and obtained nearest two genes using GREAT tool.

**Table S3** Contains list of genes that showed expression correlation with ASCL1 gene expression in TCGA primary tumor samples. Both Pearson and Spearman correlation coefficient were calculated and *p*‐values are adjusted for multiple hypothesis testing

**Table S4** Contains list of ASCL1 putative targets. Top 10% of genes that showed positive correlation (spearman coefficient) with ASCL1 expression in TCGA GBM patient samples and GBM ChIP targets were compared and listed the common genes.

**Table S5** Contains list of pathways enriched for ASCL1 putative targets. Pathway analysis was performed using ConsensuspathDB tool.

**Table S6** Contains normalized expression counts in mGBM RNA‐seq samples. Differential expression analysis was performed using DESeq2 tool.

**Table S7** Contains normalized expression counts for ASCL1 putative targets in mGBM RNA‐seq samples. Human ASCL1 putative targets were lifted over to Mouse targets using homologene database.

**Table S8** Contains normalized expression counts for genes that are differentially expressed in mGBM RNA‐seq samples with 5% FDR cut off.

## Data Availability

The data that support the findings of this study will be made available in a repository once the manuscript is accepted for publication.
