# Supplementary figures and images for: ASCL1 regulates neurodevelopmental transcription factors and cell cycle genes in brain tumors of glioma mouse models
Source: Glia. 2020 Jun 23;68(12):2613–30. doi: 10.1002/glia.23873 (PMC7587013; doi:10.1002/glia.23873)

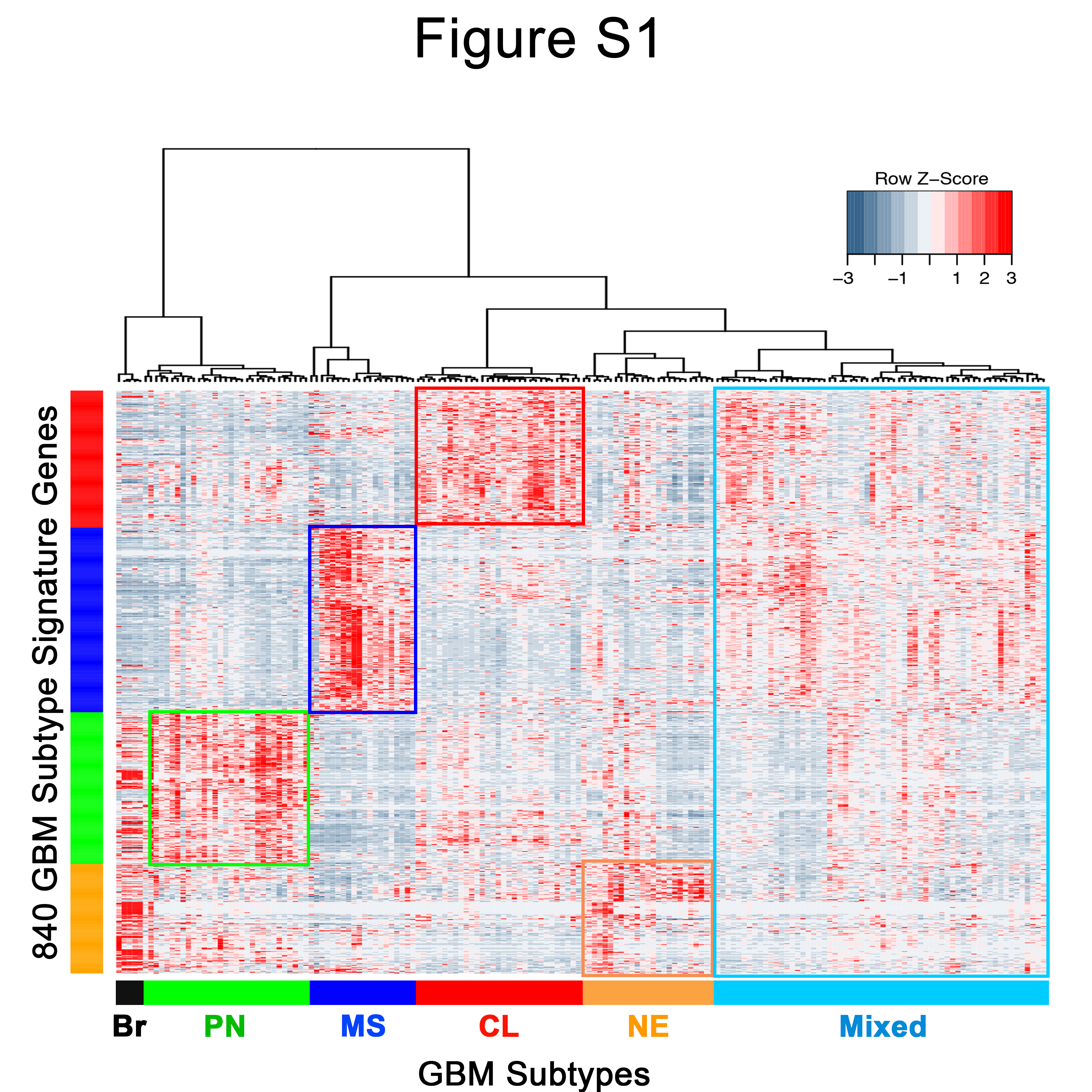

Supplement: Supplementary file 1 — Figure S1 Subtype identities of primary GBMs using RNA‐seq. RNA‐seq data of 164 TCGA Primary GBMs and 5 normal brain samples (Brennan et al., 2013). Heatmap and dendrogram using the 840 GBM Subtype Signature Genes (Verhaak et al., 2010) reveals the presence (rectangles) of four previously identified GBM subtypes (PN—proneural, MS—mesenchymal, CL—classical, NE—neural) as well as Mixed GBM group which express multiple subtype signatures. [file GLIA-68-2613-s001.tif]
